# Association between environmental lead/cadmium co-exposure in drinking water and soil and type 2 diabetes mellitus/obesity in Southern China

**DOI:** 10.3389/fpubh.2022.941922

**Published:** 2022-09-07

**Authors:** Zhi Qu, Jianli Zhou, Peisen Guo, Jingrui Wang, Panpan Wang, Limin Liu, Mengdi Wu, Peixi Wang, Nan Liu

**Affiliations:** ^1^School of Nursing, Institute of Chronic Disease Risks Assessment, Henan University, Kaifeng, China; ^2^Health Science Center, Institute of Environment and Health, South China Hospital, Shenzhen University, Shenzhen, China; ^3^College of Public Health, Zhengzhou University, Zhengzhou, China

**Keywords:** average daily dose, cadmium, lead, obesity, type 2 diabetes

## Abstract

Lead (Pb) and cadmium (Cd) in environment can be directly absorbed by drinking water and soil. However, data on human Pb and Cd exposure by drinking water and soil and its long-term consequence for type 2 diabetes mellitus (T2DM) and obesity are lacking. Our study aims to explore the association of typical heavy metals co-exposure in drinking water and soil to the community residents with T2DM and obesity indices in two cities of southern China. A cross-sectional study enrolling total 1,274 participants was performed and the local water and soil samples were collected in two communities in southern China. The average daily dose (ADD) of heavy metals was calculated to assess the exposure. The obesity indices comprise body mass index (BMI), waist-to-hip ratio (WHR) and waist circumference (WC). Binary, multiple logistic and linear regressions were employed for assessing the associations of Pb and Cd exposure with T2DM and obesity. The results showed that there weren't any significant correlations between ADDs of Pb/Cd and T2DM in community residents (all *P*s>0.05). Compared with those with 18.5 ≤ BMI <24, with 1 μg/kg bw/d ADD of Pb increase in exposure are associated with 49.2–56.1% lower likelihood of overweight. Besides, with ADDs of Pb exposure was increased by 1 μg/kg bw/d and WHR decreasing by 0.01–0.02, and WC decreasing by 2.22–4.67 cm. We speculate that Pb causes weight loss because it damages the absorption function of the gastrointestinal tract as an initial injury. 1μg/kg bw/d ADD of Cd increase is associated with 100.9% upper likelihood of low weight in Model 1. It suggests that Pb/Cd pollution in the local environment was serious and harmful to residents' health. Government should introduce relevant oversight and accountability systems to improve the prevention and management of lifestyle-related chronic diseases in the future.

## Introduction

Type 2 diabetes (T2DM) is one of the worldwide concerning public health problems. International Diabetes Federation estimated that 578 million people will have T2DM in 2030 by 25% and the number will increase by 51% (700 million) in 2045 without any interventions. Obesity is a complex and multifactorial disease with different degrees of associated cardiovascular and metabolic risk (1), and obesity was accounting for 80~85% of the risk for developing T2DM (2). Recent studies suggest that environmental exposure, especially heavy metals, could play important role in T2DM and obesity (3, 4). Exposure to toxic heavy metals has risen recently and is considered as a severe global public health problem. The types and quantities of environmental pollutants are increasing and the geographical and scale of the occurrence is gradually expanding. Among them, e-waste pollution has caused widespread concerning in Pearl River Delta Region. China is already the world's second largest producer of e-waste (2.3 million tons per year) (5). As the most common heavy metals in e-waste, exposure to lead (Pb) and cadmium (Cd) has been confirmed and affects the occurrence rate overweight/obesity and T2DM (6–8). They are frequently co-occurring in the environment and ranked in the top ten environmental chemicals of concern by environmental health agencies (1).

To date, some epidemiologic studies have reported the associations between exposure of heavy metals and T2DM/obesity, but the conclusion is not completely consistent. Some demonstrated that blood Pb/Cd level was associated with T2DM. For example, one case-control study suggested that blood Pb/Cd levels in the diabetic group were significantly higher than those in the control group (6), another study indicated that urinary Cd levels were significantly related to impaired fasting blood glucose and diabetes, and they were dose-dependent (9); while no associations of Cd exposure have been observed with T2DM (10). Data regarding Pb/Cd and obesity suggest exposure to Pb/Cd may also damage the nervous system (11–13), and obesity is associated with neurological function disorder (14).

Pb/Cd exposure to early life damage should not be ignored; for example, the risk of low birth weight (15–18) and an independent risk factor for overweight and (or) obesity in adulthood (19). Moreover, Pb with high level in dentin have been positively related to BMI in children (20). In contrast, a published cohort study demonstrated that higher levels of Pb in blood have been inversely associated with BMI in adults (8). Besides, Lee found that doubling of blood Cd concentration resulted in 26.4%~31.0% increasing of the risk of metabolic syndrome in male (7). While other study found a negative correlation between blood Cd concentration and BMI (21). Finally, animal studies have suggested that high concentration of Pb/Cd in blood accompanied with obesity may cause fast blood glucose elevation and glucose intolerance (22).

Pb and Cd in environment can be directly absorbed by drinking water and soil, it reported that 60%~80% of heavy metals exposure to the residents in industrial areas was due to contaminated food and water consumption, rather than through air pollution (https://cgspace.cgiar.org/handle/10568/46583). So far, studies on Pb/Cd co-exposure in drinking water and soil and health outcomes are limited with most of the studies coming from direct measurement of blood Pb and Cd. To the best of our knowledge, no studies have been conducted on drinking water and soil Pb exposure and T2DM/obesity in the south China population. Based on the above-mentioned evidences, the correlation between environmental Pb/Cd co-exposure in drinking water and soil and T2DM /obesity in southern China. Accordingly, the present study aimed to (1) calculate the daily Pb/Cd intakes of residents in communities in southern China through drinking water and soil and (2) evaluate the association between Pb/Cd intake (by drinking water and soil) and T2DM/obesity. Our study has a preliminary understanding of the Pb/Cd exposure level of residents and the health effects under this exposure level, which provides a further scientific reference for the damage to human health caused by heavy metal exposure. Our findings therefore could be useful for identifying the main approach and mode of the environmental exposure and the risk assessment for environmental and public health; and it also can further support and improve government's decision and policy-making for prevention and management of public health.

## Methods

### Study population and sampling sites

**Figure 1:**
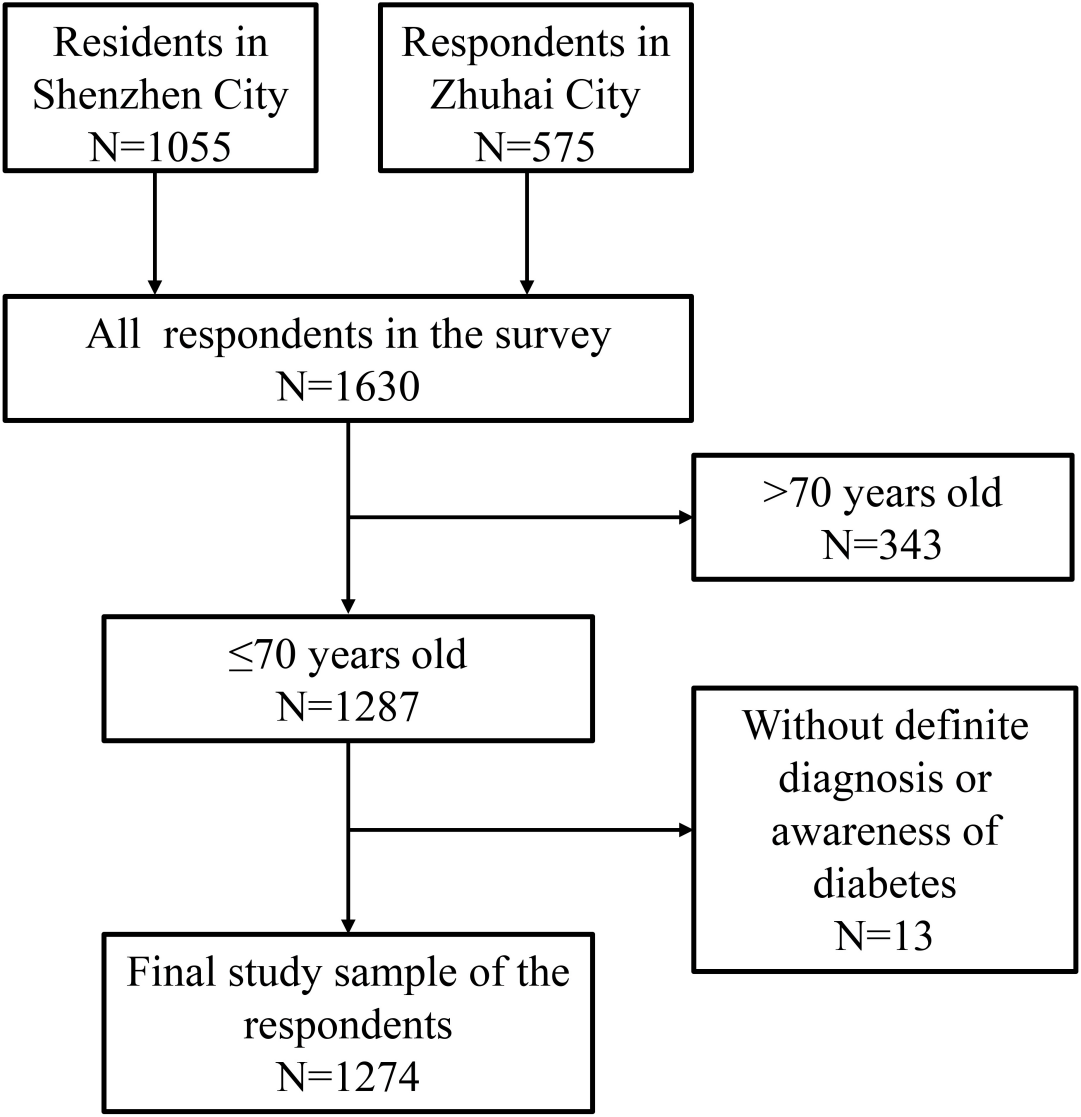
Flow chart in the selection of study subjects.

A total of 1,630 residents who live in their local community for at least 5 years were investigated by a random sampling method from Dapeng community in Shenzhen City and Hengqin community in Zhuhai City by a cross-sectional study in Guangdong Province, China, during Jul to Aug in 2018. The study was approved by the ethics committee of Health Research Specialty Committee of Guangdong Sociological Society. In our study, considering that most of the residents are middle-aged and elderly, so we recruited individuals aged 40 years or above from all respondents except the cases with incomplete questionnaire were excluded. Finally, there 1, 274 residents were included in this study. The research flow chat is described in Figure 1 which illustrates the selection of study participants. All participants were provided with informed consents.

**Figure 2:**
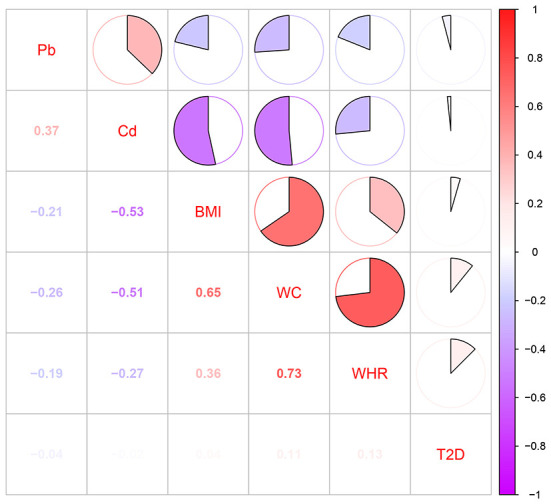
The geographical location of sampling sites. A: Shishan B: Sitang C: Xiangyang D: Santang E: Shangcun F: Hongqi G: Kuixin H: Xinwuzai I: Kuifeng J: Nanao K: Diefu L: Pengcheng M: Futang N: Huangqitang O: Nanchong

The geographical location of sampling sites as showed in Figure 2, Shenzhen and Zhuhai are located in the south of Guangdong Province, our 15 sampling sites are concentrated (nine sampling sites in Dapeng community in Shenzhen City (nine drinking water samples and nine soil samples) and six sampling sites in Hengqin community in Zhuhai City (six drinking water samples and six soil samples)) in the southeast of Shenzhen and Zhuhai.

### Data collection and measurements

All surveys were conducted and the data were collected by face-to-face interviews and physical measurements by interview groups, and each small group was composed of at least two local healthcare staffs and using structured and standard questionnaires. Initially, each group was accompanied by a supervisor to ensure that the interviews were properly conducted. The investigators have received professional trainings. We collected information by questionnaire survey about demographic characteristics (sex, age, marital status, educational level, per capita monthly income), lifestyle risk factors (smoking status, drinking status, depression or anxiety, frequency of exercises per week, days of eating fresh vegetables per week, days of eating fresh fruits per week) and medical history (family history of T2DM, hypertension state and diabetic state). Smoking status is divided into three categories: never smoke, former smoke and current smoke, which, respectively means those who reported smoking fewer than 100 cigarettes in their lifetime; ever smoked at least 100 cigarettes in their lifetime but haven't currently smoked; and smoked at least 100 cigarettes and currently smoked some days or every day. The average number of alcoholic drinks consumed per day in the past year was calculated based on the reported frequency and average number of drinks on a consumption day (23). The hypertension state and diabetic state were self-report by doctor's diagnosis. Anthropometric data including body weight, height, waist circumference (WC) and hip circumference (HC) were measured according to NIH guidelines.

BMI = body weight (kg)/height^2^ (cm^2^). According to the standard of China, BMI < 18.5 is defined as chronic malnutrition and lean; 18.5 ≤ BMI <24 is normal weight; 24 ≤ BMI <28 is overweight; and 28 ≤ BMI is considered as obese. WHR= waist circumference (cm)/hip circumference (cm). WC ≥90 cm and ≥ 85 cm are abdominal obesity for male and female, respectively (24). The obesity indices comprise generalized obesity indices (BMI) and abdominal obesity indices (WHR and WC) (25).

### Quality control of the survey

All data were well-checked by the well-trained staffs that have inquired and measured the involved participants. In addition, a special quality monitoring team supervised and reviewed all the completed questionnaires to ensure the authenticity and reliability.

### Environmental sample collection and analysis

Drinking water samples were collected according to the standard examination methods for drinking water-collection and preservation of water samples (GBT5750.2-2006) (26). Soil samples were collected according to soil quality-Guidelines on sampling techniques (http://www.china-soilquality.com/biaozhundongtai/2019/0109/114.html). Concentrations of Pb/Cd in drinking water and soil were measured using inductively coupled plasma mass spectrometry (ICP-MS) in Public Monitoring Center for Agro-product of Guangdong Academy of Agricultural Sciences, Guangzhou, P. R. China. Water samples were collected from the main source of drinking water for the local population; and soil samples were collected from nearby fields where vegetables were grown, the heavy metals in water and soil may be ingested by water and food intake. Hence, those collected water and soil samples might represent the exposure levels of the local population.

### Exposure assessment for Pb/Cd

The exposure dose of adults was assessed by using the method developed by the US Environmental Protection Agency (EPA) for measuring heavy metals exposure risk in drinking water and soil. Among all the different routes of exposure, the oral route remains the least documented (27). As we all know, oral intake is the most important route of exposure to heavy metals in drinking water and soil, in this study we only consider the oral intake exposure to water and ingestion exposure to soil in the common communities in southern China without heavy air pollution and occupational exposure. The ADD exposure to heavy metals was calculated to assess the exposure using the following formulas from EPA protocol (28, 29) as Equation (1).


(1) $$\begin{array}{r} {\text{ADD}}_{\text{d}-\text{w}}=\left({\text{C}}_{\text{d}-\text{w}}\text{×}{\text{IR}}_{\text{d}-\text{w}}\text{×}{\text{EF}}_{\text{d}-\text{w}}\text{×}{\text{ED}}_{\text{d}-\text{w}}\right)/\left(\text{BW×}{\text{AT}}_{\text{d}-\text{w}}\right). \end{array}$$

The calculation model of ADD exposure to Pb or Cd in soil is exhibited in Equation (2).


(2) $$\begin{array}{r} {\text{ADD}}_{\text{s}}=\left({\text{C}}_{\text{s}}\text{×}{\text{IR}}_{\text{s}}\text{×}{\text{EF}}_{\text{s}}\text{×}{\text{ED}}_{\text{s}}\text{×}10^{-3}\right)/\left(\text{BW×}{\text{AT}}_{\text{s}}\right) \end{array}$$

Where ADD is the average daily intake via ingestion of drinking water or soil [μg/(kg·d)]; C_d−w_ and C_s_ are the heavy metals concentration in drinking water and soil, respectively (μg/L, μg/kg); IR_d−w_ is the daily amount of drinking water for adults (L/d); EF_d−w_ is the frequency of exposure (d/y); ED_d−w_ is the duration of exposure (y); AT_d−w_ is the average exposure time (d). IR_s_ is the ingestion rates of soil (mg/d); EF_s_ is the exposure frequency of soil (d/y); ED_s_ is the exposure duration of soil (y); AT_s_ is the average time of dose (d); BW is the body weight (kg). The calculation parameters (30, 31) were demonstrated in Supplementary Table S1. The total intakes [μg/(kg·d)] of heavy metals via ingestion pathways were added from by ADDs of water and soil.

### Statistical analysis

Medium and quartile were use to describe the central trend. Binary, multiple and linear logistic regression analyses were employed to evaluate the correlation between ADDs of Pb/Cd and T2DM, ADDs of Pb/Cd and BMI, ADDs of Pb/Cd and WHR/WC, respectively. The effect of each heavy metal was evaluated separately. Adjusted confounding factors include socio-demographic characteristics and lifestyle factors. The three regression Models appointed as Model 1, 2 and 3 for the adjusted regressions for socio-demographic characteristics, lifestyle factors and the both, respectively. The categorical and continuous confounding variables in the Models were fitted as dummy variables and the raw data. All statistical analyses were performed using SPSS version 22.0 (SPSS Inc., Chicago, IL, USA) and R Core Team (2020). R: A language and environment for statistical computing (http://www.R-project.org/), with a two-sided *P*-value < 0.05 considered as statistically significant.

## Results

### Incidence rate of T2DM and obesity in Southern China

The incidence rates of T2DM, obesity and abdominal general obesity in China and southern China were illustrated in Supplementary Table S2. It suggested that the incidence rate of T2DM and general obesity in Southern China were slightly lower than that in China level while the incidence rate of abdominal obesity was higher than national level of China, suggesting that in our study, although many people have normal BMI, their WC has exceeded the normal level.

### Concentration of heavy metals in soil/water samples and calculation of total intakes

**Figure 3:**
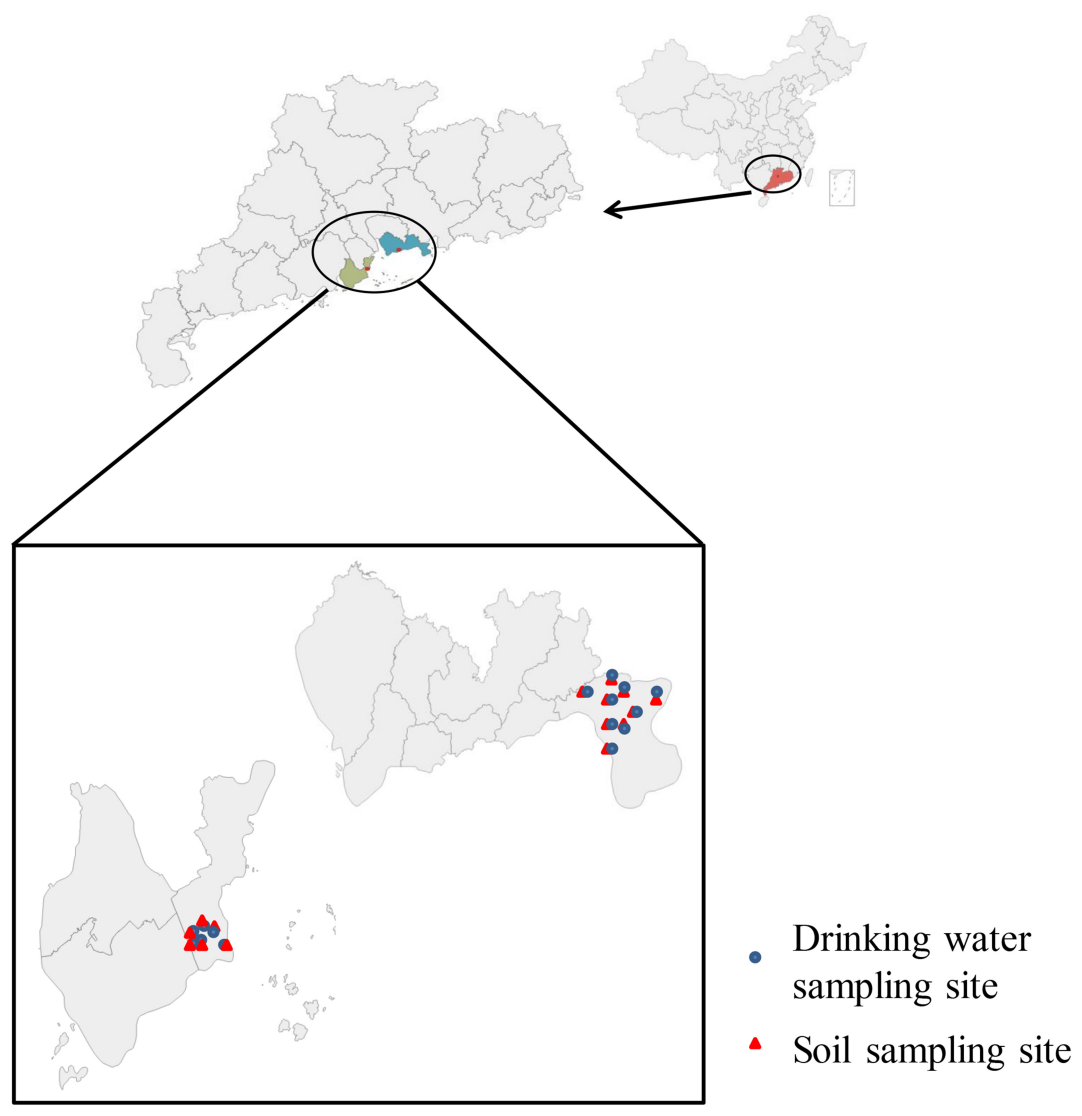
Pearson correlation coefficients between heavy metals (Pb and Cd) and obesity indices (BMI, WC, and WHR).

The concentrations of Pb/Cd in each sampling region were demonstrated in Table 1. The maximum concentrations of Pb and Cd detected in the drinking water were 0.022 and 0.043 mg L^−1^, respectively. The maximum concentrations of them in soil samples were 75.600 and 7.860 mg kg^−1^. Exposure assessment for heavy metals was exhibited in Supplementary Figure S2. It highlighted that the range of ADDs of Pb and Cd via drinking water was 0.01–1.05 and 0.03–2.21 μg/kg bw/d, respectively (Supplementary Figure S1A). The ranges of ADD of Pb and Cd via soil were 0.02–0.22 and almost close to 0 (The maximum exposure level was 0.01 μg/kg bw/d) μg/kg bw/d, respectively (Supplementary Figure S1B). The range and medium (the number in the parentheses) of total ADDs of Pb and Cd were 0.03–1.21 (0.101), 0.03–2.21 (0.053) μg/kg bw/d, respectively (Supplementary Figure S1C), and we found the exposure dose of most of the residents were below the median levels. The ADDs of Pb and Cd grouped by the residents' demographic sociological characteristics and lifestyle factors were exhibited in Supplementary Table S3. Spearman correlations for Pb/Cd exposure, T2DM, and each of the obesity indices (BMI, WC and WHR) were demonstrated in Figure 3. The correlation between BMI and WC is *r*_s_ = 0.65. The correlation between WC and WHR is *r*_s_ =0.73. BMI and WC; WC and WHR have high correlation (*r*_s_ >|0.6|), and the variables with high correlation are not placed in the same model.

**Table 1:** Concentrations of Pb and Cd in water and soil of different sampling areas.

**Location**	**Sample**	**Concentrations of**		**Location**	**Sample**	**Concentrations of**	
	**type**	**heavy metals** ^**a**^			**type**	**heavy metals** ^**a**^	
		**Pb**	**Cd**			**Pb**	**Cd**
A	Water	0.012	0.016	I	Water	ND	ND
	Soil	48.620	3.450		Soil	13.50	0.176
B	Water	0.014	0.043	J	Water	0.001	ND
	Soil	39.140	7.860		Soil	19.30	0.158
C	Water	0.001	ND	K	Water	0.016	ND
	Soil	25.480	5.180		Soil	62.50	0.682
D	Water	0.002	0.005	L	Water	0.013	ND
	Soil	37.250	0.180		Soil	88.60	7.200
E	Water	0.012	0.026	M	Water	0.001	ND
	Soil	28.230	2.840		Soil	15.200	0.154
F	Water	0.001	ND	N	Water	0.001	ND
	Soil	24.580	0.470		Soil	13.900	0.065
G	Water	ND	ND	O	Water	0.002	ND
	Soil	25.200	0.074		Soil	11.800	0.057
H	Water	0.022	ND				
	Soil	75.600	0.043				

a The unit of heavy metal concentration is mg L^−1^ in water and mg kg^−1^ in soil; ND, not detected.

### Effects of ADDs of Pb/Cd on T2DM in community residents

**Figure 4:**
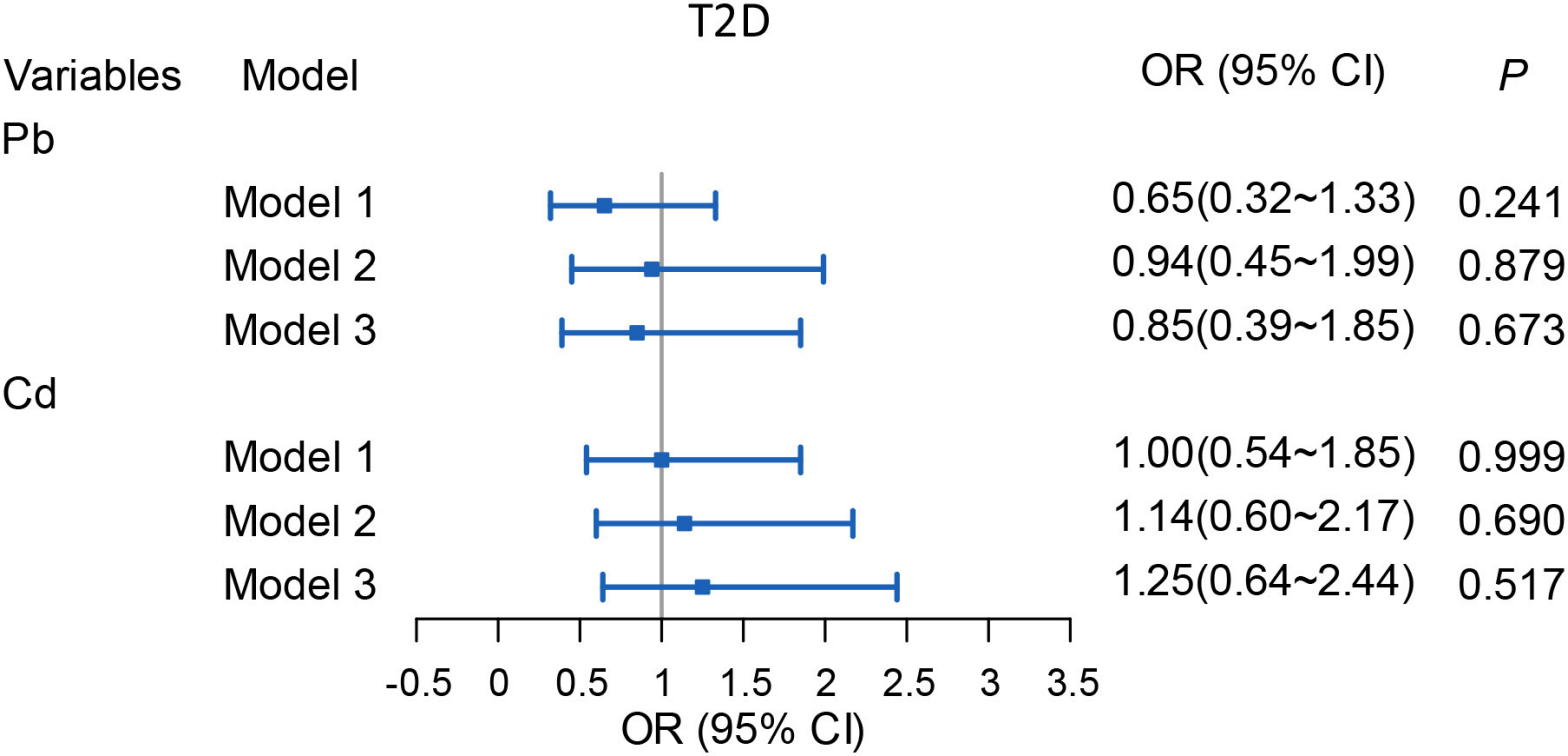
Associations of ADDs [μg/(kg·d)] of Pb/Cd with T2DM among the total subjects. Model 1: The adjusted values for per capita monthly income, sex, age, education level and marital status, family history of T2DM. Model 2: The adjusted values for smoking, drinking, hypertension, weekly exercise time, grade of BMI, WHR, and weekly intake of fresh vegetables/fruits, anxiety or depression state and the stillness and sitting time. Model 3: The adjusted values for the covariates in Model 1 and Model 2. Pb, Total intake of Pb; Cd. Total intake of Cd.

Binary logistic regression analysis was employed for evaluating the effect of ADDs of Pb/Cd exposure on T2DM of community residents. However, we haven't found any correlations between ADDs of Pb/Cd exposure and T2DM in community residents in all the three models (Figure 4).

### Effects of the ADDs of Pb/Cd to BMI in community residents

**Figure 5:**
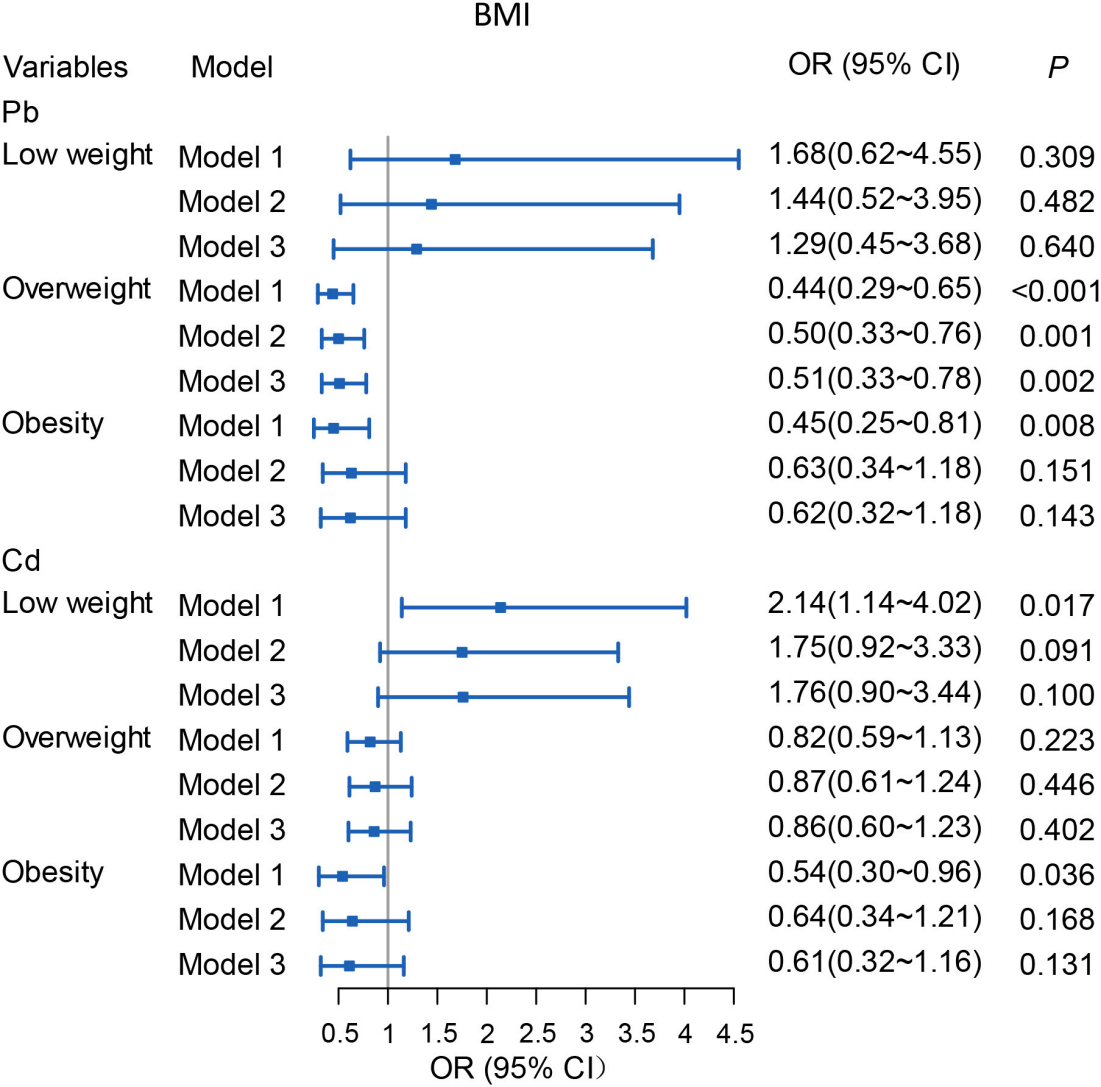
Associations of ADDs [μg/(kg·d)] of Pb/Cd with BMI among total subjects. Model 1: The adjusted values for per capita monthly income, sex, age, education level and marital status. Model 2: The adjusted values for smoking, drinking, T2DM, hypertension, WHR, weekly exercise times, grade of BMI, and weekly intake of fresh vegetables/fruits; anxiety or depression state and stillness and sitting time. Model 3: The adjusted values for the covariates in Model 1 and Model 2. Reference level: Normal weight (18.5 ≤ BMI <24). Pb, Total intake of Pb; Cd, Total intake of Cd.

Multiple logistic regression analysis was employed to evaluate the effect of ADDs of Pb/Cd exposure to BMI of community residents in Figure 5. Compared with the residents with 18.5 ≤ BMI <24, we found that with 1 μg/kg bw/d ADD of Cd increase in exposure is associated with 100.9% upper likelihood of low weight in Model 1, with OR (95% CI): 2.01 (1.08–3.73). However, there is no association in Model 2 and 3. We found that compared with those with 18.5 ≤ BMI <24, 1 μg/kg bw/d ADD of Pb increase in exposure is associated with 49.2–56.1% lower likelihood of overweight, with ORs (95% CIs) were as follows: Model 1: OR (95% CI): 0.44 (0.30–0.66); Model 2: OR (95% CI): 0.50 (0.33–0.76); Model 3: OR (95% CI): 0.51 (0.33–0.78). Our research suggested that compared with the residents with 18.5 ≤ BMI <24, the residents with 1 μg/kg bw/d ADD of Pb increase in exposure was associated with 53.5% lower likelihood of obese in Model 1, with OR and 95% CI were 0.47 and 0.26–0.84, but there is no association in Model 2 and 3, which means higher ADD of Pb makes subjects less likely to be overweight.

### Effects of ADDs of Pb/Cd to WHR and WC in community residents

**Figure 6:**
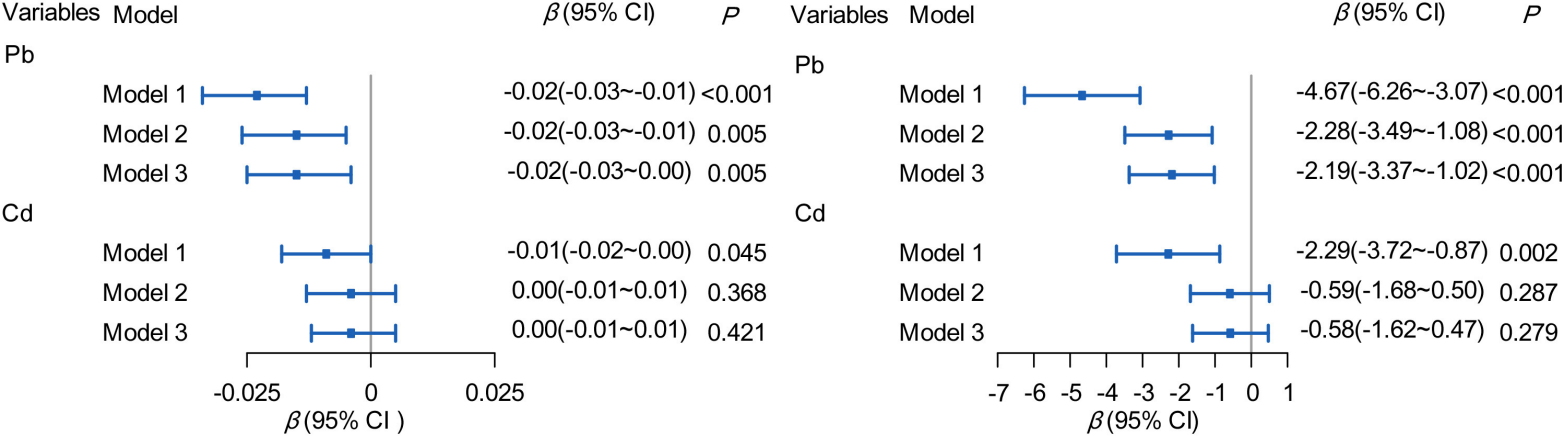
Association of ADDs [μg/(kg·d)] of Pb/Cd with WHR and WC. Model 1: The adjusted for per capita monthly income, sex, age, education level and marital status. Model 2: The adjusted for smoking, drinking, T2DM, hypertension, weekly exercise times, grade of BMI, and weekly intake of fresh vegetables/fruits, anxiety or depression state and stillness and sitting time. Model 3: The adjusted for the covariates in Model 1 and Model 2. Pb, Total intake of Pb; Cd, Total intake of Cd.

Linear regression analysis was used to analyze the effects of ADDs of Pb/Cd exposure on WHR and WC of the community residents. We found that there was a negative correlation between ADD of Pb and WHR in all the models. With the increasing of ADDs of Pb exposure by 1 μg/kg bw/d, WHR was decreased by 0.01–0.02, the parameters of β and 95% CI were as follows: Model 1: β (95% CI): −0.02 (−0.03 to −0.01); Model 2: β = −0.015 (−0.03 to −0.00); Model 3: β = −0.014, (−0.02 to −0.00). However, there was no correlation between ADD of Cd exposure and WHR of the community residents. Pb exposure was found an effect on WC, and when ADD of Pb exposure was increased by 1 μg/(kg·d) with WC decreasing by 4.655 cm (β = −4.66, 95% CI: −6.24 to −3.07), 2.31 cm (β = −2.31, 95% CI: −3.50 to −1.11) and 2.22 cm (β = −2.22, 95% CI: −3.38 to −1.06) in Model 1, 2, and 3, respectively. Besides, ADDs of Cd exposure was increased by 1 μg/(kg·d) with WC decreasing by 1.84 cm (β = −1.84,95% CI:−3.21 to −0.47) in Model 1, but there is no association in Model 2 and 3. All the details were shown in Figure 6.

## Discussion

T2DM and obesity have been prevalent in Chinese population, especially in the middle-aged and elderly people (32). In this work, we explored the association of typical heavy metals, mainly Pb/Cd co-exposure in drinking water and soil to the community residents with T2DM and obesity indices in southern China. Our results indicate that abdominal obesity rate (35.4%) is higher than the national level of China (31.5%). Abdominal body fat can increase the risk of diabetes and carries greater risk of developing future cardiovascular events than peripheral or gluteofemoral obesity (33).

Our goal was to control for as many other factors as possible that contribute to T2DM and obesity, and then assess the association of Pb/Cd in drinking water and soil with these diseases, rather than the influence of demographic characteristics and health-related behaviors on disease. In this work, we found both Pb and Cd are not associated with T2DM. Compared with the residents with 18.5 ≤ BMI <24, the possibility of overweight was reduced by 49.2–56.1% when the exposure to Pb increased by 1 μg/kg bw/d ADD. Pb was negatively correlated with WHR and WC.

WHO emphasizes the dietary exposure corresponding to an increase in systolic blood pressure of 1 mmHg (0.13 kPa) was estimated to be 1.3 (5th to 95th percentiles 0.6–28) μg/kg bw/d (https://apps.who.int/food-additives-contaminants-jecfa-database/chemical.aspx?chemID = 3,511). In our work, the highest level of daily Pb exposure in this study was 1.21 μg/kg bw/d (Supplementary Figure S1), although it was slightly lower than 1.3 μg/kg bw/d, the Committee of WHO concluded that the provisional tolerable weekly intake (PTWI) could no longer be considered health protective, and it was withdrawn (https://apps.who.int/food-additives-contaminants-jecfa-database/chemical.aspx?chemID = 3,511). Our maximum exposure level of Cd was 66.3 μg/kg bw/m (2.21 μg/kg bw/d ×30), which is more than twice the PTMI recommended by WHO (25 μg/kg bw/m) (https://apps.who.int/food-additives-contaminants-jecfa-database/chemical.aspx?chemID = 1,376). Therefore, Pb and Cd pollution in the local environment should be paid sufficient attention.

There have been studies on the effects of Pb and Cd exposure on T2DM and obesity, while most of them focused on assessing the levels of heavy metals in blood and urine (7, 10, 23). In our investigation, we found the heavy metals especially Pb and Cd coexistence in local soil and water background were exceed the standard of China due to some electronic processing plants and e-waste incineration sites near the communities in Shenzhen and Zhuhai where the survey was conducted.

As the rapid economic development in southern China of Shenzhen and Zhuhai, the ecological environment, water sources and soils in the areas have been seriously contaminated, and the tap-water in some areas was extracted from the polluted water sources directly. The effects have continued for about 20–30 y to the present owing to the extensive approach to dealing with e-waste in the Pearl River Delta. Since 1.5–2 L/d of water consuming is required for a person, drinking water is a major way of exposure to heavy metals, and previous studies have proved soil ingestion is an important intake route of Pb/Cd exposure (34). Therefore, the relationship between Pb/Cd intake through drinking water and soil and the diseases are still worthy of further studies.

The correlations of the epidemiological study on heavy metal exposure and diabetes are not entirely consistent. It reported that in an investigation of 238 diabetic patients and 196 age-matched non-diabetic patients, the average blood Pb/Cd levels in the diabetic group were significantly higher than those in the control group (6). Another study suggested that a significant non-linear association between urinary Cd levels and prediabetes (35). In our previous study, we found concentrations of blood Mn showed a significant association with prevalence of metabolic syndrome only in age 30–49 and non-linear associations were observed of four heavy metals (Pb, Cd, mercury and manganese) in blood with the risks of MetS among all participants and in specific age and gender groups by restricted cubic splines logistic regression (36). However, there are also some inconsistent relevance between Cd and T2DM (3, 37). Besides, in the general Korean population, it implied that blood Pb and Cd have no significant relationship with diabetes (4). Compared with exposure to lower concentrations of Pb and Cd, our results suggested that Pb/Cd was not associated with T2DM exposure with higher exposing concentrations (Figure 3). Animal experiments showed that the rats were fed with Pb acetate (0.05% w/v) resulted in poor glucose tolerance (22). Besides, another epidemiological report found that daily intake of 6.5–16.25 μg Cd through drinking water could induce hyperglycemia and change the lipid metabolism (3). Hence, we speculate that the level of ADDs of Pb/Cd exposure in our study may not be sufficient to induce the clinical symptom of T2DM, however it might contribute to some physical damage such as poor glucose tolerance or hyperglycemia. On the other hand, owing to the negative association between Pb/Cd exposure and obesity, the association between Pb/Cd exposure and T2DM has been correspondingly reduced as obesity is an intermediate variable in the etiological chain of T2DM.

The relationship of the epidemiological study on heavy metal exposure and obesity are not also entirely consistent. One report in Boston suggested that chronic Pb exposure in childhood might result in obesity that persisting into adulthood (20). While others studies demonstrated that the concentrations of Pb in blood were negatively correlated with BMI and WC (8, 21, 23). Our finding suggested that with 1 μg/kg bw/d ADD of Pb exposure increase, the likelihood of overweight to normal weight (18.5 ≤ BMI <24) decreased 49.2–56.1% (Figure 5), and the WHR decreased 0.01–0.02, or the waist measurement decreased by 2.22–4.67 cm, which is consistent with the previous studies (8, 21, 23). Regarding the weight loss caused by Pb exposure, the following mechanisms can explain it. On the one hand, an animal study has revealed that most of the orally intake of Pb remains in the intestinal mucosa, and a small amount of Pb was absorbed by the gastrointestinal tract, the unabsorbed Pb may affect the balance of the digestive tract and intestinal environment (38). Therefore, we speculate that Pb causes weight loss because it damages the absorption function of the gastrointestinal tract. On the other hand, animal studies found that lead can depress the appetite so that induce a reduction in body weight (39). Some studies demonstrated that the concentrations of Cd in blood were negatively correlated with BMI and WC (8, 21, 23), while our study showed the opposite results. There are also some studies that were consistent with our findings (40). About the inconsistent conclusions, it still requires further and strong evidences.

The global pandemic of obesity, over-nutritional symptoms and climate change caused by a combination of economic, social and environmental factors. Currently, the consumption of meat and ultra-processed foods which with high sugar, fat, and calories. The greenhouse gas emissions, sedentary lifestyles, unhealthy travel patterns, and environmental changes all contribute to the global pandemic. To help bolster the battle of the bulge, government issued a guideline recently to implement the Healthy China initiative, i.e., an outline for the “Healthy China 2030,” which encourages people to adopt healthier lifestyles and diets. The guideline sets specific targets for reducing and limiting daily salt intake to <5 g, cooking oil intake to between 25 and 30 g and sugar intake to <25 g by 2030, etc.

Many studies have shown that such advertising can influence children's food preferences and intake (41). As a result, the WHO advises governments and food manufacturers to limit the advertising and promotion of unhealthy foods to children (42). In China, however, there is almost no limitation for food advertising. China should enact legal regulation of food advertising to children to limit the advertising of junk food, such as sugary drinks and high-calorie snacks, aimed at children.

Our study also has some advantages and disadvantages. The advantage is as follows: our study assessed residents' exposure to heavy metals through drinking water and soil, which is easier to obtain samples and reduction of expenditure compared with direct measurement of heavy metals in blood. However, our research has some limitations. Firstly, the fasting blood glucose and insulin levels haven't included in this survey as they are the indicators of pre-diabetes. Secondly, in fact, the evaluation system factors, such as pollutant concentration distribution, per capita drinking water consumption and the exposure frequency, etc. are uncertain, and it is difficult to accurately reflect the total personal exposure. The sources of exposure to heavy metals are not only from drinking water and soil sources mentioned in this study, but also from dietary and air, so our study might underestimate the exposure of Pb/Cd.

## Conclusion

As a cross-sectional study, we have evaluated the association between Pb/Cd co-exposure in drinking water and soil and T2DM/Obesity in southern China. It helps to understand the distribution characteristics of disease or health status. In general, increase of ADD of Pb exposure lower the likelihood of becoming overweight; ADD of Pb was negatively correlated with WHR and WC. As the results are still contradictory to previous studies, large-sample and multiple-area surveys on the relationship between living environment and long-term impact health effects are necessary.

## Data availability statement

The original contributions presented in the study are included in the article/Supplementary material, further inquiries can be directed to the corresponding author/s.

## Ethics statement

The studies involving human participants were reviewed and approved by the Ethics Committee of Health Research Specialty Committee of Guangdong Sociological Society. The patients/participants provided their written informed consent to participate in this study.

## Author contributions

PeW and NL were involved in the administrative support, conception and design of the manuscript, data interpretation, reviewed, and revised the manuscript. JZ was involved in the conception and design of the manuscript, collection and assembly of the data, data analysis, and drafted the initial manuscript. ZQ, PG, JW, and PaW were involved in the collection and assembly of data and drafted the initial manuscript. LL and MW were involved in the conception and design of the manuscript, reviewed, and revised the manuscript. PeW and NL were involved in critically reviewed the manuscript for important intellectual content. All the authors contributed to the article and approved the submitted version of the manuscript.

## Funding

The authors gratefully acknowledge the financial support by the National Natural Science Foundation of China (No. 81872584), Natural Science Foundation of Shenzhen (No. JCYJ20210324093211030), the Medical Scientific Research Foundation of Guangdong Province (No. A2020490), Key R&D and Promotion Projects of Henan Province (No. 222102310587), Military Logistics Research Project (No. CKJ20J031), and Interdisciplinary Research for First-class Discipline Construction Project of Henan University (No. 2019YLXKJC04).

## Conflict of interest

The authors declare that the research was conducted in the absence of any commercial or financial relationships that could be construed as a potential conflict of interest.

## Publisher's note

All claims expressed in this article are solely those of the authors and do not necessarily represent those of their affiliated organizations, or those of the publisher, the editors and the reviewers. Any product that may be evaluated in this article, or claim that may be made by its manufacturer, is not guaranteed or endorsed by the publisher.
