# Supplementary material for: Association between environmental lead/cadmium co-exposure in drinking water and soil and type 2 diabetes mellitus/obesity in Southern China
Source: Front Public Health. 2022 Sep 7;10:941922. doi: 10.3389/fpubh.2022.941922 (PMC9489910; doi:10.3389/fpubh.2022.941922)
Supplement: Supplementary file 1 [file Data_Sheet_1.docx]

**Supplementary Information**

**Association between environmental lead/cadmium co-exposure and** **type 2** **diabetes/obesity in southern China**

Zhi Qu ^1, 2^†, Jianli Zhou ^1, 2^†, Peisen Guo ^2, 3^, Jingrui Wang ^1^, Panpan Wang ^2, 3^, Limin Liu ^2, 3^, Mengdi Wu ^2, 3^, Peixi Wang ^1, *^, Nan Liu ^1, 2, 3 *^**
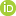
**

^1^ Institute of Chronic Disease Risks Assessment, School of Nursing, Henan University, Kaifeng, 475004, P. R. China

^2^ Institute of Environment and Health, South China Hospital, Health Science Center, Shenzhen University, Shenzhen, 518116, P. R. China.

^3^ College of Public Health, Zhengzhou University, Zhengzhou, 540001, P. R. China.

*Corresponding authors:

**Peixi Wang**

[peixi001@163.com](mailto:peixi001@163.com)

**Nan Liu**

[13688869875@163.com](mailto:13688869875@163.com)

orcid.org/0000-0002-8895-3169

**Contents**

**Figure S1** Environmental exposure assessment for Pb and Cd[μg/(kg🞗d)].

The ADDs of (a) Drinking water; (b) Soils; (c) Total.

**Table S1** Parameters of exposure assessment on Pb/Cd in environment

**Table S2** ADDs of Pb and Cd grouped by demographic sociological characteristics and lifestyle factors behaviors

**Supplementary Figures**

**
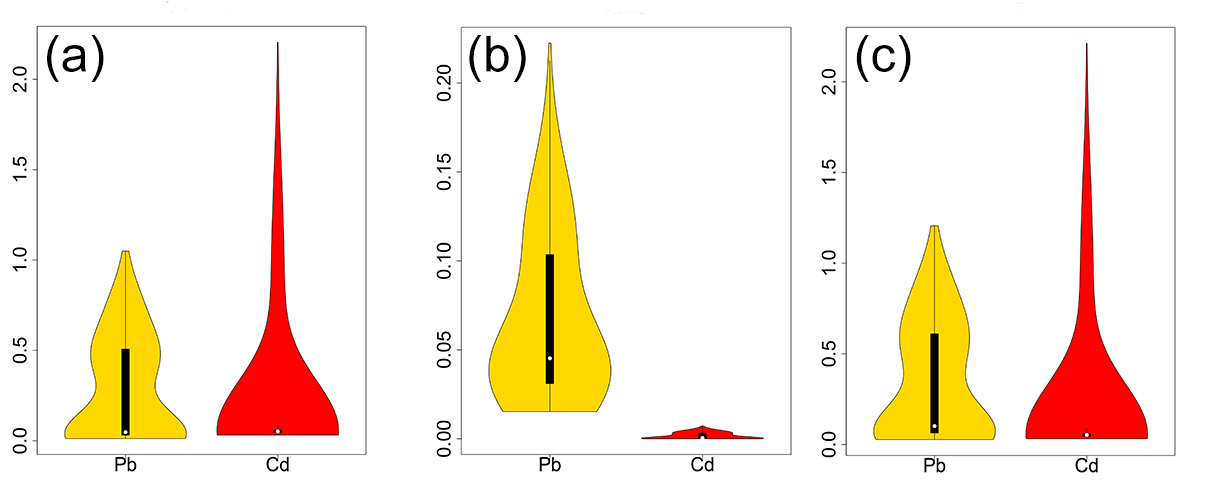
**

**Figure S1** Environmental exposure assessment for Pb and Cd[μg/(kg🞗d)].

The ADDs of (a) Drinking water; (b) Soils; (c) Total.

**Supplementary Tables**

**Table S1** Parameters of exposure assessment on heavy metals in environment

| **Item** | **Parameter** | **Value** | **Unit** | **Data Sources** |
| --- | --- | --- | --- | --- |
| C_d-w_ | Heavy metals concentration in drinking water | - | ug/L | This work |
| IR_d-w_ | Daily intake volume of drinking water | 2 | L/d | Ref ([You et al., 2011](#_ENREF_1)) |
| EF_d-w_ | Exposure frequency of drinking water | 365 | d/y | Ref ([You et al., 2011](#_ENREF_1)) |
| ED_d-w_ | Exposure duration of drinking water | 31.3 (Pb)/73(Cd) | y | Ref ([You et al., 2011](#_ENREF_1)) |
| AT_d-w_ | Average exposure time | 31. 3 y ×360 d (Pb)/73 y ×365 d (Cd) | d | Ref ([You et al., 2011](#_ENREF_1)) |
| C_s_ | Heavy metals concentration in soil | - | μg/kg | This work |
| IR_s_ | Ingestion rates | 100 | mg/d | Ref ([Zhuo et al., 2019](#_ENREF_2)) |
| EF_s_ | Exposure frequency of soil | 350 | d/y | Ref ([Zhuo et al., 2019](#_ENREF_2)) |
| ED_s_ | Exposure duration of soil | 26 | y | Ref ([Zhuo et al., 2019](#_ENREF_2)) |
| BW | Body weight | - | kg | Investigation and survey (individual) |
| ATs | Average exposure time | ED×365 (Pb)/70×365 (Cd) | d | Ref ([Zhuo et al., 2019](#_ENREF_2)) |

**Table S2** **The Incidence rate of T2DM and obesity in China and Southern China**

| Area | Incidence rate (%) | | |
| --- | --- | --- | --- |
|  | **T2DM** | **General obesity** | **Abdominal obesity** |
| China* | 9.4 ^a^ | 14.0 ^b^ | 31.5 ^b^ |
| Southern China | 7.1 | 11.6 | 35.4 |

***** According to the Chinese criteria, general obesity was defined as BMI≥28 kg/m^2^ and abdominal obesity as WC≥90 and 85 cm for male and female.

^a^ T2DM data source is coming from WHO, <https://www.who.int/diabetes/country-profiles/zh/>.

^b^ The general obesity and abdominal obesity data source is coming from reference 26.

**Table S3 ADDs of Pb and Cd grouped by demographic sociological characteristics and** **lifestyle factors behaviors**

| **Item** | **Property** | **Intake of Pb (μg/L)** | | | | |  | **Intake of Cd (μg/L)** | | | | |
| --- | --- | --- | --- | --- | --- | --- | --- | --- | --- | --- | --- | --- |
|  |  | P25 | P50 | | P75 | |  | P25 | P50 | | P75 | |
| **Sex** |  |  | |  | |  |  |  | |  | |  |
|  | Male | 0.06 | | 0.08 | | 0.47 |  | 0.04 | | 0.05 | | 0.06 |
|  | Female | 0.07 | | 0.44 | | 0.67 |  | 0.05 | | 0.05 | | 0.06 |
| **Age** | 40-50 | 0.06 | | 0.09 | | 0.58 |  | 0.05 | | 0.05 | | 0.06 |
|  | 51-60 | 0.06 | | 0.09 | | 0.58 |  | 0.05 | | 0.05 | | 0.06 |
|  | 61-70 | 0.06 | | 0.42 | | 0.68 |  | 0.05 | | 0.05 | | 0.06 |
| **Marital status** | Unmarried | 0.06 | | 0.08 | | 0.51 |  | 0.05 | | 0.05 | | 0.07 |
|  | Married | 0.06 | | 0.11 | | 0.61 |  | 0.05 | | 0.05 | | 0.06 |
|  | Divorced | 0.06 | | 0.07 | | 0.1 |  | 0.04 | | 0.05 | | 0.05 |
|  | Widowed | 0.06 | | 0.08 | | 0.77 |  | 0.05 | | 0.06 | | 0.06 |
| **Educational level** | Illiteracy and semiliterate | 0.07 | | 0.49 | | 0.73 |  | 0.05 | | 0.05 | | 0.06 |
|  | Primary school | 0.06 | | 0.39 | | 0.62 |  | 0.05 | | 0.05 | | 0.06 |
|  | Junior high school | 0.06 | | 0.09 | | 0.58 |  | 0.05 | | 0.05 | | 0.06 |
|  | High school | 0.06 | | 0.08 | | 0.59 |  | 0.05 | | 0.05 | | 0.06 |
|  | College and above | 0.06 | | 0.09 | | 0.56 |  | 0.04 | | 0.05 | | 0.06 |
| **Per capita monthly income** | <4999 | 0.06 | | 0.10 | | 0.61 |  | 0.05 | | 0.05 | | 0.06 |
|  | ≧5000 | 0.06 | | 0.08 | | 0.55 |  | 0.05 | | 0.05 | | 0.06 |
|  | Unknown | 0.06 | | 0.40 | | 0.64 |  | 0.05 | | 0.05 | | 0.06 |
| **Smoking status** | Never smoke | 0.06 | | 0.11 | | 0.64 |  | 0.05 | | 0.05 | | 0.06 |
|  | Current smoking | 0.06 | | 0.08 | | 0.48 |  | 0.04 | | 0.05 | | 0.07 |
|  | Quit smoking | 0.05 | | 0.11 | | 0.45 |  | 0.05 | | 0.05 | | 0.77 |
| **Drinking status** | Never drinking | 0.06 | | 0.11 | | 0.63 |  | 0.05 | | 0.05 | | 0.06 |
|  | Current drinking | 0.06 | | 0.08 | | 0.5 |  | 0.04 | | 0.05 | | 0.06 |
|  | Quit | 0.06 | | 0.13 | | 0.63 |  | 0.05 | | 0.05 | | 0.07 |
| **Family history of T2D** | No | 0.06 | | 0.11 | | 0.62 |  | 0.05 | | 0.05 | | 0.06 |
|  | Yes | 0.06 | | 0.08 | | 0.56 |  | 0.04 | | 0.05 | | 0.06 |
| **Depression or anxiety** | No | 0.06 | | 0.10 | | 0.61 |  | 0.05 | | 0.05 | | 0.06 |
|  | Yes | 0.06 | | 0.41 | | 0.70 |  | 0.04 | | 0.05 | | 0.06 |
| **frequency of exercises per week** | 0-3 | 0.06 | | 0.10 | | 0.63 |  | 0.05 | | 0.05 | | 0.06 |
|  | 4-7 | 0.07 | | 0.10 | | 0.57 |  | 0.05 | | 0.05 | | 0.07 |
| **Days of eating fresh vegetables per week** | 0-3 | 0.06 | | 0.40 | | 0.6 |  | 0.05 | | 0.05 | | 0.06 |
|  | 4-7 | 0.06 | | 0.10 | | 0.61 |  | 0.05 | | 0.05 | | 0.06 |
| **Days of eating fresh fruits per week** | 0-3 | 0.07 | | 0.42 | | 0.69 |  | 0.05 | | 0.05 | | 0.07 |
|  | 4-7 | 0.06 | | 0.09 | | 0.6 |  | 0.05 | | 0.05 | | 0.06 |
| **BMI level** | BMI <18.5 | 0.09 | | 0.13 | | 0.77 |  | 0.07 | | 0.07 | | 0.08 |
|  | 18.5≤BMI<24 | 0.07 | | 0.14 | | 0.67 |  | 0.05 | | 0.06 | | 0.06 |
|  | 24≤BMI<28 | 0.06 | | 0.08 | | 0.54 |  | 0.04 | | 0.05 | | 0.05 |
|  | BMI≥28 | 0.05 | | 0.34 | | 0.5 |  | 0.04 | | 0.04 | | 0.05 |
| **Hypertension state** | No | 0.06 | | 0.10 | | 0.62 |  | 0.05 | | 0.05 | | 0.06 |
|  | Yes | 0.06 | | 0.09 | | 0.59 |  | 0.04 | | 0.05 | | 0.06 |
| **Diabetic state** | No | 0.06 | | 0.10 | | 0.62 |  | 0.05 | | 0.05 | | 0.06 |
|  | Yes | 0.06 | | 0.10 | | 0.56 |  | 0.05 | | 0.05 | | 0.06 |
| **WC** ≥**90 cm (male)/** ≥ **85 cm(female)** | No | 0.07 | | 0.10 | | 0.64 |  | 0.05 | | 0.06 | | 0.07 |
|  | Yes | 0.05 | | 0.09 | | 0.54 |  | 0.04 | | 0.05 | | 0.05 |
| **WHR** ≥**0.9 (male)/** ≥**0.8(female)** | No | 0.07 | | 0.09 | | 0.56 |  | 0.05 | | 0.05 | | 0.07 |
|  | Yes | 0.06 | | 0.11 | | 0.62 |  | 0.05 | | 0.05 | | 0.06 |

SD: standard error

**References**

You, H., Pang, Z., Liang, Y., Lin, Y., 2011. Health risk assessment of heavy metals in drinking water in a certain district of Foshan City. S Chin J Prev Med 37, 32-36.

Zhuo, H., Fu, S., Liu, H., Song, H., Ren, L., 2019. Soil heavy metal contamination and health risk assessment associated with development zones in Shandong, China. Environmental Science and Pollution Research 26, 30016-30028.
